# Food environment and diabetes mellitus in South Asia: A geospatial analysis of health outcome data

**DOI:** 10.1371/journal.pmed.1003970

**Published:** 2022-04-26

**Authors:** Dian Kusuma, Petya Atanasova, Elisa Pineda, Ranjit Mohan Anjana, Laksara De Silva, Abu AM Hanif, Mehedi Hasan, Md. Mokbul Hossain, Susantha Indrawansa, Deepal Jayamanne, Sujeet Jha, Anuradhani Kasturiratne, Prasad Katulanda, Khadija I Khawaja, Balachandran Kumarendran, Malay K Mridha, Vindya Rajakaruna, John C Chambers, Gary Frost, Franco Sassi, Marisa Miraldo

**Affiliations:** 1 Centre for Health Economics Policy Innovation, Imperial College Business School, London, United Kingdom; 2 School of Public Health, Imperial College London, United Kingdom; 3 Madras Diabetes Research Foundation, Chennai, India; 4 Faculty of Medicine, University of Colombo, Colombo, Sri Lanka; 5 Centre for Non-Communicable Diseases and Nutrition, BRAC James P Grant School of Public Health, BRAC University, Dhaka, Bangladesh; 6 Faculty of Medicine, University of Kelaniya, Ragama, Sri Lanka; 7 Max Healthcare, New Delhi, India; 8 Services Institute of Medical Sciences, Lahore, Pakistan; 9 Faculty of Medicine, University of Jaffna, Kokkuvil, Sri Lanka; 10 Lee Kong Chian School of Medicine, Nanyang Technological University, Singapore, Singapore; 11 Faculty of Medicine, Imperial College London, London, United Kingdom; 12 Department of Economics and Public Policy, Imperial College Business School, London, United Kingdom; Carolina Population Center, UNITED STATES

## Abstract

**Background:**

The global epidemic of type 2 diabetes mellitus (T2DM) renders its prevention a major public health priority. A key risk factor of diabetes is obesity and poor diets. Food environments have been found to influence people’s diets and obesity, positing they may play a role in the prevalence of diabetes. Yet, there is scant evidence on the role they may play in the context of low- and middle-income countries (LMICs). We examined the associations of food environments on T2DM among adults and its heterogeneity by income and sex.

**Methods and findings:**

We linked individual health outcome data of 12,167 individuals from a network of health surveillance sites (the South Asia Biobank) to the density and proximity of food outlets geolocated around their homes from environment mapping survey data collected between 2018 and 2020 in Bangladesh and Sri Lanka. Density was defined as share of food outlets within 300 m from study participant’s home, and proximity was defined as having at least 1 outlet within 100 m from home. The outcome variables include fasting blood glucose level, high blood glucose, and self-reported diagnosed diabetes. Control variables included demographics, socioeconomic status (SES), health status, healthcare utilization, and physical activities. Data were analyzed in ArcMap 10.3 and STATA 15.1. A higher share of fast-food restaurants (FFR) was associated with a 9.21 mg/dl blood glucose increase (95% CI: 0.17, 18.24; *p* < 0.05). Having at least 1 FFR in the proximity was associated with 2.14 mg/dl blood glucose increase (CI: 0.55, 3.72; *p* < 0.01). A 1% increase in the share of FFR near an individual’s home was associated with 8% increase in the probability of being clinically diagnosed as a diabetic (average marginal effects (AMEs): 0.08; CI: 0.02, 0.14; *p* < 0.05). Having at least 1 FFR near home was associated with 16% (odds ratio [OR]: 1.16; CI: 1.01, 1.33; *p* < 0.05) and 19% (OR: 1.19; CI: 1.03, 1.38; *p* < 0.05) increases in the odds of higher blood glucose levels and diagnosed diabetes, respectively. The positive association between FFR density and blood glucose level was stronger among women than men, but the association between FFR proximity and blood glucose level was stronger among men as well as among those with higher incomes. One of the study’s key limitations is that we measured exposure to food environments around residency geolocation; however, participants may source their meals elsewhere.

**Conclusions:**

Our results suggest that the exposure to fast-food outlets may have a detrimental impact on the risk of T2DM, especially among females and higher-income earners. Policies should target changes in the food environments to promote better diets and prevent T2DM.

## Abstract 2

Dian Kusuma and colleagues investigate the associations between exposure to the density and proximity of healthy and unhealthy food outlets and diabetes in Bangladesh and Sri Lanka.

## Author summary

**Why was this study done?:**

- With a global prevalence of 9% (463 million people) in 2019, the widespread epidemic of diabetes mellitus renders the prevention of this condition a major priority for public health.
- Creating health-enabling environments that promote healthy eating and physical activity is needed to reduce the number of people suffering from diabetes.
- Food environments influence risk factors for type 2 diabetes mellitus (T2DM), but this evidence is understudied among low- and middle-income countries (LMICs).

**What did the researchers do and find?:**

- We examined the associations between the exposure to the density and proximity of healthy and unhealthy food outlets and diabetes (i.e., fasting blood glucose level, high blood glucose, and diagnosed diabetes) using the South Asia Biobank and environment mapping data for 12,167 adults collected between 2018 and 2020 for Bangladesh and Sri Lanka.
- We found that a higher share of fast-food outlets was associated with a 9.21 mg/dl blood glucose increase. Also, a 1% increase in the share of fast-food outlets was associated with an 8% increase in the probability of being diagnosed with diabetes. Having at least 1 fast-food retailer in the proximity of one’s home was associated with 2.14 mg/dl blood glucose increase.
- While the association between the density of fast-food retailers and blood glucose level was stronger among women than men, density of fast-food restaurants (FFRs) increased the likelihood of being diagnosed with diabetes for both men and women. The association between proximity to these outlets and blood glucose level was stronger among men.
- Both density and proximity of fast-food outlets were associated with diabetes outcomes among high-income earners. Density of FFRs was associated with increased probability of being diagnosed with diabetes for low-income earners, although the effect size was smaller than for high-income earners.

**What do these findings mean?:**

- The exposure to fast-food outlets may have a detrimental impact on the risk of diabetes, especially among females and those with higher income.
- Policies should target changes in the food environments to promote better diets and prevent diabetes.

## Introduction

With a global prevalence of 9% (463 million people) in 2019, the widespread epidemic of type 2 diabetes mellitus (T2DM) renders its prevention a major public health priority [1]. Although, historically, T2DM was considered a disease confined to countries of affluence, recent estimates suggest that 80% of the 463 million people with T2DM now live in low- and middle-income countries (LMICs) [2]. South Asia is particularly affected by T2DM, where the highest number of deaths were attributable to diabetes under the age of 60 years (working age) in 2019. The projected increase in T2DM prevalence for 2030 in the region is substantially higher (74%) in comparison to that in Europe (15%) [1].

In 2020, the Lancet Diabetes Commission recommended creating health-enabling environments that promote healthy eating and physical activity to reduce the number of people suffering from T2DM [3]. This highlights the importance of the role the food environment plays in driving diabetes prevalence. A cohort study of the United Kingdom Biobank using a sample of 502,625 participants found that the density of ready-to-eat food establishments (pubs and bars, restaurants and cafeterias, and fast food or hot and cold takeaway outlets) within a 1-km street catchment area was associated with higher odds of T2DM [4]. Another cohort study of more than 4.5 million participants from Sweden found that the density of health-harming food outlets (fast-food outlets, convenience stores, bars, and pubs) within 1-km buffer from an individual’s home was associated with a greater likelihood of T2DM prevalence and incidence [5]. Moreover, den Braver and colleagues conducted a systematic review of 109 eligible studies and found that living in an urban residence was associated with higher T2DM risk. However, it found that evidence of an association between food environment with T2DM risk remains inconsistent [6].

In this study, we assessed the role food environment plays on diabetes outcomes among adults in 2 understudied LMICs, Bangladesh and Sri Lanka, where the prevalence of diabetes has been growing rapidly. In 2020, the prevalence of diabetes was 8.1% and 8.7% in Bangladesh and Sri Lanka, which correspond to, respectively, an estimated 8.4 million and 1.2 million total cases of diabetes among adults [7]. These trends may be driven by an increase in the prevalence of risk factors for diabetes such as obesity and poor diets. The prevalence of obesity was 25.9% in 2018 and 29.3% in 2014 among adults in Bangladesh and Sri Lanka, respectively [8]. Moreover, evidence from Sri Lanka indicates that in 2013 only 3.5% of adults consumed the recommended 5 portions of fruits and vegetables (FV). In contrast, they consumed over 14 portions of starch and 3.5 portions of added sugars daily [9]. Further, consumption patterns and food sourcing have been found to vary across sex and the different income strata. For example, higher-income individuals are more likely to source food from conveniently located stores that might offer more product variety at higher prices, whereas lower-income groups are more likely to travel longer distances to stationary and/or mobile markets where the food is relatively cheap and readily available [10]. Food environments have been found to influence diets and obesity and therefore may be instrumental for the prevention of diabetes in these countries [3,11,12]. Yet, evidence on the association of the food environment and diabetes is limited, and, to our knowledge, no study has assessed this association in South Asia.

Most existing evidence is for high-income countries (e.g., United States, United Kingdom, Australia, Japan, and Sweden) and measure exposure to food environments using geographic information systems (GISs) data of food environments (e.g., density or proximity of food outlets) [6]. There are some contributions in LMICs (e.g., Thailand, Malaysia, India, Sri Lanka, Ghana, Nigeria, and Uganda), but exposure to food environments tends to be captured with crude metrics such as residency in urban/rural areas, which are imperfect in capturing the different elements of the environment as well as individual exposure to those environments. Second, previous studies focused on self-reported diagnosed T2DM status, which may have been subject to recall bias and did not included the undiagnosed populations, as shown by the UK Biobank study [4]. Thus, our study aims to fill the evidence gap by investigating the associations between the density and proximity of food environment and T2DM in Bangladesh and Sri Lanka, using a rich characterization of exposure to healthy and unhealthy food environments in the neighborhood of peoples’ homes. Our research question is whether food environment is associated with T2DM. We hypothesize that healthy elements of the food environments are negatively associated with T2DM, while unhealthy elements of the food environment are positively associated with T2DM.

## Methods

### Study design and sample

We carried out a novel approach of examining the associations between exposure to food-built environments and T2DM. We linked individual health outcome data of 12,167 individuals from our network of health surveillance sites (the South Asia Biobank) to the density and proximity of food outlets geolocated around their homes from our environment mapping surveys collected between 2018 and 2020 in Bangladesh and Sri Lanka.

### South asia biobank

The South Asia Biobank is a comprehensive biobank of South Asian individuals, established to identify the risk factors and their complex interactions underlying the development of T2DM, cardiovascular disease, and other chronic diseases in South Asians. It is a cross-sectional investigation in Bangladesh, India, Pakistan, and Sri Lanka with data collected between November 2018 and March 2020. Data include participants’ demographic, lifestyle, clinical, environmental, and phenotypic characteristics and biological samples. In each country, national administrative data were used to select rural and urban surveillance sites. One district was randomly selected from each of the major administrative divisions or provinces, from which one subdistrict was randomly chosen. One or more community clinics (or urban dispensary) within each subdistrict were randomly selected and the ward where those community clinics located were surveillance sites. All eligible residents in a surveillance site were invited to participate. In the recruitment of participants, governmental census data and available household listings were used, together with house-to-house visits by local research teams, to identify the residents. Further detail on the sample and data can be found in Song and colleagues [13].

### Outcome variables

There were 3 outcome variables in our analyses: diagnosed diabetes, fasting blood glucose level, and high blood glucose. First, diagnosed diabetes was a binary variable with a value of 1 if one reported ever been told by health worker having a raised blood sugar (i.e., diabetes) or currently takes medications (e.g., insulin) and 0 if otherwise. An interviewer-administered health and lifestyle questionnaire was used to collect information on diagnosed diabetes, along with other behavioral risk factors (smoking, alcohol use, physical activity, and consumption of FV), medical history, medications, and socioeconomic status (SES). Although diagnosed diabetes was self-reported, individuals were asked whether they had been told by their doctor they had diabetes or if they were on prescribed medication (e.g., insulin) rather than whether they thought they had diabetes. This, together with the fact that data collection took place in a healthcare setting, may mitigate some of the issues associated with self-reported data. However, to mitigate biases from self-reported data, we complement the analyses with 2 objectively measured outcomes, namely fasting blood glucose level (mg/dl), which was a continuous variable obtained from blood samples taken by trained data collectors in each surveillance site in the South Asia Biobank study and high blood glucose, which was a binary variable with a value of 1 if the fasting blood glucose was 126 mg/dl and above and 0 if otherwise. Fasting glucose was measured by point of care tests. Equipment, protocols, and training were standardized across surveillance sites [13].

### Environmental mapping

In each surveillance site in Bangladesh and Sri Lanka, an environmental mapping was conducted to characterize the built environment in terms of the number of food outlets. We created a list of questions including geolocations, type of food outlets, and select items sold (e.g., FV, confectionary, and fast food). To ensure comprehensiveness, we adapted the questions and data collection procedure from the International Network for Food and Obesity/NCDs Research, Monitoring and Action Support (www.informas.org) and Johns Hopkins University’s Maryland Food Systems Map (https://mdfoodsystemmap.org). We then discussed with local research teams to include local-specific food retailer types and food items sold. We included all questions in an online questionnaire using KoBoToolbox application (www.kobotoolbox.org) as the study instrument. Prior to data collection, local research teams in each country were trained in administering the instrument on smartphones or tablets. During October 2018 to August 2020, 6-person research teams conducted ground truth data collection surveys, by systematically covering all streets within surveillance sites on foot, following a map in which the site boundary was previously defined [13]. The team examined and recorded the presence of any food retailer within each site. To ensure that all streets and neighborhoods in surveillance sites were covered, we reviewed the map of food outlets on KoBoToolbox website and Google Maps together with each country team. Also, we deployed quality control checks by sending a second team to the sites to conduct spot checks in randomly selected areas within each site to ensure that all areas were appropriately covered and all relevant food outlets were recorded [14]. Our mapping collected data on geolocations (latitude and longitude) of supermarkets, corner stores (including small grocery and convenient stores), mobile food carts, stationary food carts, and restaurants. Mobile food outlets (stalls, carts, vans, and bikes) are important source of both healthy (e.g., fish and FV) and unhealthy food (e.g., snacks and sugary drinks) especially in LMICs—sample images from our mapping are in **S1 Fig**. Therefore, it was important to capture their availability. Because they are mobile, teams would visit the sites both in the mornings and afternoons, and control checks were performed in days and times that differed from the original data collection timings to ensure the presence of these outlets would be captured in the data.

### Food environment exposures

To our knowledge, there is no food outlet classification in South Asia, and, therefore, we followed the international classification of healthy and unhealthy food outlets as described in the Retail Food Environment Index [15] and the North American Industry Classification System (www.census.gov/naics). We created 5 categories for food outlets: (1) fast-food restaurants, including international as well as domestic fast-food restaurants where people can purchase sweetened beverages and speedy, ready-to-eat food that is highly processed and high in calories and thus considered unhealthy; (2) supermarkets, self-service shop selling fresh FV and other healthy foods, therefore considered healthy; (3) corner stores, small shop selling foods; (4) mobile carts, temporary structure that is readily moveable; and (5) stationary carts, moveable structure but occupies a specific location [16,17]. Since we did not observe what is sold in each outlet, in reporting the results, we followed the literature in classifying supermarkets as healthy food outlets and fast-food restaurants as unhealthy food outlets [17]. Even though supermarkets sell a range of healthy and unhealthy products, in international classifications, these are described as healthy due to the fact that they are more likely to provide healthier options compared to fast-food restaurants (FFRs) or corner stores [15]. Therefore, for supermarkets and FFRs, we adopt the terminology of healthy and unhealthy food outlets as in international classifications for the purpose of formulating our research hypotheses, but we remain agnostic to the direction of the association of each food retailer on diabetes outcomes [15]. For stationary carts and mobile carts, there is no consensus in the literature on their classification, and, therefore, we remain agnostic, in terms of research hypotheses, on whether they are healthy or unhealthy.

We calculated the density of and proximity to fast-food outlets, supermarkets, corner stores, mobile carts, and stationary carts, which together encompasses what we here refer to as the food environment. Food environment geolocation data were merged with individual-level data to characterize the density of different food outlet types within 300-m buffer around each participant’s home. We counted the total number of each food outlet and defined density as the share of each food outlet type relative to all food outlets within the 300-m buffer. Also, we defined the proximity of food outlets as having at least 1 food outlet type within 100-m of a participant’s home address—variable definitions are summarized in **S1 Table**. Similar distances have been used in the literature and enables capturing more variation in terms of individual exposure to the food environments [18,19]. Geospatial analyses were conducted on ArcMap 10.3.

### Data analysis

For fasting blood glucose level (a continuous variable), we employed ordinary least squares (OLS) multivariate regressions to assess its association with food environment. In reporting the estimates, we used the OLS coefficients for both density and proximity as the measure of food environment exposure. For high blood glucose and diagnosed diabetes (binary variables), we employed logistic regressions to test the associations with food environment. In reporting the estimates, we report average marginal effects (AMEs) for density (a continuous variable) and adjusted odds ratios (AOR) for proximity (a binary variable), as the measures of food environment exposure. In addition to these models using the entire sample, we also stratify by sex and income. The low- and high-income strata were defined based on whether each individual had an income of above (henceforth high income) or below (henceforth low income) the median income among the sample in each country.

We adjusted for individual-level covariates based on previous literature [20]. They included demographic characteristics (i.e., sex, age, country, marital status, and religion), SES (i.e., paid employment, school years, income, and household composition), health status measured (i.e., self-assessed health), healthcare utilization (i.e., receiving advice from health workers to reduce the consumption of products high in fat and sugary beverages, to increase daily intake of FV, to lose weight, or to increase physical activity), and physical activity habits (i.e., weekly minutes of vigorous or moderate physical activity spent at work, home or recreational facilities, and walking or cycling as a mode of transportation). We control for religion because, in the context of South Asia, studies have reported that religious affiliations could significantly impact individuals’ dietary patterns, physical activity, and, ultimately, may impact the risk of diabetes and other noncommunicable diseases (NCDs). For example, evidence suggests that Muslim populations tend to have higher prevalence of NCDs and indicated that Muslims consume more deep-fried and processed foods and spend less time in physical activity (especially Muslim women) compared to non-Muslim, even when controlled for education and income status [21].

Income was reported in USD after being adjusted for purchasing power parity (PPP) for comparability between the 2 countries and was deflated using 2018 prices. Regressions also included surveillance site as the fixed effects to control for site-specific time invariant cofounders. The analyses were well powered using a sample of 12,167 participants and conducted in STATA 15.1. More details of model specifications are provided in **S1 Text**, and estimates from unadjusted regressions are provided in **S2 Table**. This study is reported as per the Strengthening the Reporting of Observational Studies in Epidemiology (STROBE) guideline—see provided in **S3 Table**.

The study did not have a published protocol, but it had a planned analysis in the research proposal and research plans submitted to the funder (**S2 Text**). Also, the study had a (nonwritten) internal plan for the analyses, which were assessed by the Global Health Research Unit (GHRU) Steering Committee. We have not deviated from the planned analyses during the execution of the analyses, nor during the review process.

Research approval was obtained from the Imperial College London Research Ethics Committee (reference: 18IC4698) and local institutional review boards in each of the participating countries (Bangladesh [BRAC University] and Sri Lanka [University of Kelaniya and University of Colombo]).

## Results

For the total sample of *N* = 12,167, which included participants from Bangladesh (*N* = 8,534) and Sri Lanka (*N* = 3,633), the average age was 45.5 (14.4 SD) years, school years were 6.42 (4.84 SD), and monthly income was 700.73 USD (**Table 1**). From the total, 59.7% (49.1 SD) were females, and 43.9% had a paid employment. Regarding the food environment (panels B and C), the average share of FFR within 300 m of a resident’s home address was 7.78% (9.74% SD). Corner stores and supermarket shares were 50.79% (33.20% SD) and 0.88% (3.37% SD), respectively. The share of FFR was higher in Bangladesh, while the share of corner stores and supermarkets were higher in Sri Lanka. The share of supermarkets was zero in rural areas, indicating that all supermarkets in our sample were in urban sites. For diabetes mellitus, defined as high blood glucose (>126 mg/dl) and diagnosed diabetes, the mean fasting blood glucose level was 102.92 (33.86 SD) mg/dl. The proportion of high blood glucose (>126 mg/dl) was 11.13% (31.46% SD) and that of diagnosed diabetes was 11.90% (32.39% SD). Diabetes mellitus levels were higher among females and higher-income participants (**Table 1**).

**Table 1:** Descriptive statistics of sample characteristics.

VARIABLES	Total *N* = 12,167	Male *N* = 4,897	Female *N* = 7,256	Lower income *N* = 6,031	Higher income *N* = 6,048	Sri Lanka *N* = 3,633	Bangladesh *N* = 8,534
**(A) General characteristics**							
**Female, *n* (%)**	7,254 (59.71%)	0%	100%	61.75%	57.66%	69.12%	55.7%
**Age years, mean (SD)**	45.47 (14.42)	47.03	44.41	47.05	43.87	49.68	43.67
**Married, *n* (%)**	10,587 (87.01%)	90.06%	84.99%	86.69%	88.61%	81.15%	89.51%
**Urban, *n* (%)**	7,207 (59.23%)	56.30%	61.21%	56.61%	61.89%	81.53%	49.74%
**Religion, *n* (%)**							
Buddhist	2,601 (21.38%)	16.15%	24.88%	19.43%	23.63%	71.59%	0%
Christian	368 (3.02%)	2.14%	3.62%	2.70%	3.39%	10.13%	0%
Hindu	927 (7.62%)	8.13%	7.28%	9.67%	5.69%	11.01%	6.18%
Muslim	8,165 (67.11%)	72.76%	63.31%	68.00%	67.20%	5.28%	93.43%
Other religion	106 (0.87%)	0.82%	0.91%	0.20%	0.10%	1.98%	0.40%
**School years, mean (SD)**	6.42 (4.84)	6.47	6.31	5.26	7.57	9.82	4.90
**Income $PPP, mean (SD)**	700.73 (1,860.13)	741.42	672.67	318.94	1,081.44	901.23	616.51
**Employed, *n* (%)**	5,307 (43.94%)	85.56%	15.81%	41.62%	46.25%	40.69%	45.30%
**Number of adults in household, mean (SD)**	3.09 (1.35)	3.17	3.04	2.81	3.37	3.21	3.04
**Self-assessed health, *n* (%)**							
Poor	1,762 (14.59%)	11.89%	16.41%	17.01%	12.17%	7.22%	17.68%
Fair	4,130 (34.19%)	34.55%	33.93%	33.33%	35.05%	30.34%	35.81%
Good	5,587 (46.25%)	47.80%	45.22%	45.40%	47.11%	51.30%	44.13%
Very good	479 (3.97%)	4.69%	3.48%	3.28%	4.65%	8.87%	1.90%
Excellent	121 (1.00%)	1.07%	0.96%	0.98%	1.03%	2.27%	0.47%
**Physical activity mins/w, mean (SD)**							
Vigorous	397.98 (1,050.53)	753.19	159.01	492.66	309.35	126.63	513.49
Moderate	793.76 (1,043.02)	580.30	938.07	855.411	743.84	455.08	937.95
Transport week	152.26 (253.58)	217.49	108.23	163.37	143.39	128.11	162.54
**Received advice from health providers on, *n* (%)**							
Increasing FV	4,837 (40.04%)	36.55%	42.45%	37.42%	42.66%	32.52%	43.20%
Reducing fat content in diet	3,727 (30.86%)	28.18%	32.69%	27.89%	33.81%	36.69%	28.40%
Increasing PA	2,502 (20.71%)	17.67%	22.80%	17.68%	23.74%	31.07%	16.36%
Losing weight	2,582 (21.38%)	16.95%	24.38%	17.44%	25.30%	30.48%	17.55%
Reducing sugary beverages	2,213 (18.32%)	16.47%	19.56%	16.32%	20.32%	29.44%	13.65%
**(B) Density food outlet (%)**							
FFR share	7.78 (9.75)	7.74	7.79	7.36	8.20	6.56	8.29
Supermarket share	0.88 (3.37)	0.79	0.93	0.70	1.06	1.99	0.40
Corner store share	50.79 (33.20)	50.47	50.99	48.20	53.48	53.55	49.61
Mobile cart share	1.57 (5.16)	1.52	1.60	1.51	1.63	1.75	1.49
Stationary cart share	11.76 (18.39)	11.99	11.59	11.85	11.71	9.96	12.52
**(C) Proximity food outlet (%)**							
FFR	18.53 (38.86)	18.32	18.67	14.43	22.69	13.87	20.52
Supermarket	22.29 (41.62)	4.59	5.17	2.59	7.29	6.17	4.41
Corner store	50.70 (50.00)	50.50	50.80	46.05	55.47	47.51	52.06
Mobile cart	7.59 (26.49)	7.15	7.90	5.77	9.42	7.40	7.68
Stationary cart	4.93 (21.65)	21.58	22.75	19.88	24.69	21.88	22.46
**(D) Outcome variables**							
Fasting blood glucose (mg/dl), mean (SD)	102.92 (33.86)	101.71	103.75	101.78	103.97	106.36	101.47
High blood glucose (1 = 126+ mg/dl), *n* (%)	1,343 (11.13%)	10.27%	11.71%	9.77%	12.38%	16.00%	9.08%
Diagnosed diabetes (1 = diagnosed), *n* (%)	1,438 (11.90%)	11.37%	12.26%	10.46%	13.34%	19.79%	8.59%

Note: Mean (SD) = mean (standard deviation) of total sample (Sri Lanka and Bangladesh). Low income and high income were defined as below or above the median income in USD PPP. PA vigorous activity = minutes of vigorous physical activity per week at work, home, or recreational centers; PA moderate activity = moderate physical activity per week at work, home, or recreational centers; PA transport week = minutes spent walking or bicycling as a mode of transportation per week. Health utilization = receiving advice on increasing FV or reducing fat content in diet, or increasing physical activity, or losing weight, or reducing the consumption of sugary beverages. Density or share of FFRs, corner store, stationary cart, mobile cart, and supermarket = the number of each food outlet per total number of food outlets within 300 m of a resident’s home address. Proximity of FFR, corner store, stationary cart, mobile cart, and supermarket = 1 if having at least 1 outlet within 100 m of a resident’s home address and 0 if otherwise.

FFR, fast-food restaurant; FV, fruits and vegetables; PA, physical activity; PPP, purchasing power parity.

**Fig 1:**
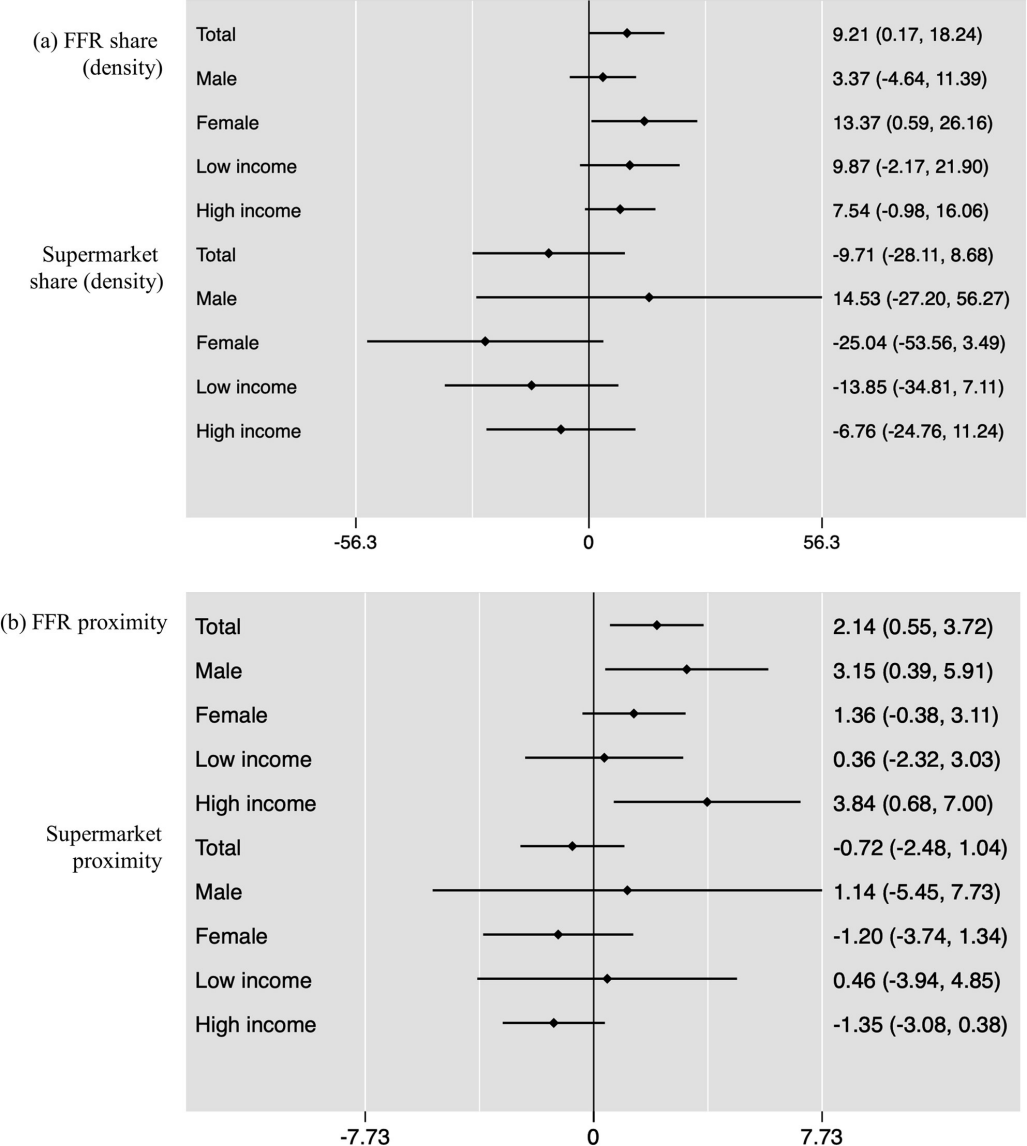
Associations between fasting blood glucose level and density (a) and proximity (b) of food outlets. Note: The values show OLS regression coefficients; 95% CIs in brackets. Density = the number of each food outlet per total number of food outlets within 300 m of a resident’s home address. Proximity = 1 if having at least 1 outlet within 100 m of a resident’s home address and 0 if otherwise. FFR, fast-food restaurant; OLS, ordinary least squares.

Regarding the associations between fasting blood glucose level and the density and proximity of food outlets, no statistically significant results were found for the share of supermarket, corner stores, stationary carts, or mobile carts (**Fig 1**, **Tables 2 and 3**). However, a higher FFR share was associated with a 9.21 mg/dl blood glucose increase (95% CI, 0.17, 18.24; *p* < 0.05). Similarly, having at least 1 FFR in the proximity (i.e., within 100 m) of one’s home was associated with 2.14 mg/dl blood glucose increase (CI: 0.55, 3.72; *p* < 0.01). When stratifying by sex, FFR densities/shares were associated with a greater blood glucose increase in females (β = 13.37; CI: 0.59, 26.16; *p* < 0.05), while FFR proximities were associated with a higher blood glucose increase in males (β = 3.15; CI: 0.39, 5.91; *p* < 0.05) and in those with higher income (β = 3.84; CI: 0.68, 7.00; *p* < 0.05).

**Table 2:** Associations between food outlet density and fasting blood glucose (OLS coefficients), high blood glucose, and diagnosed DM (AMEs).

**A. Blood glucose level**	**Total**	**Male**	**Female**	**Low income**	**High income**	**Sri Lanka**	**Bangladesh**
FFR share	9.21*	3.37	13.37*	9.87	7.54	0.32	10.40
	(0.17 to 18.24)	(−4.64 to 11.39)	(0.59 to 26.16)	(−2.17 to 21.90)	(−0.98 to 16.06)	(−10.15 to 10.79)	(−0.77 to 21.57)
Supermarket share	−9.71	14.53	−25.04	−13.85	−6.76	−2.91	−47.52**
	(−28.11 to 8.68)	(−27.20 to 56.27)	(−53.56 to 3.49)	(−34.81 to 7.11)	(−24.76 to 11.24)	(−18.70 to 12.89)	(−52.28 to −42.75)
Corner store share	−0.48	−3.22	1.35	1.84	−3.21	−3.28	0.28
	(−4.52 to 3.57)	(−7.37 to 0.92)	(−3.07 to 5.76)	(−1.45 to 5.14)	(−9.00 to 2.59)	(−11.05 to 4.48)	(−4.10 to 4.66)
Mobile cart share	−7.86	−0.96	−12.93	1.08	−17.54**	−37.81**	−1.75
	(−24.18 to 8.47)	(−11.27 to 9.34)	(−34.86 to 8.99)	(−21.08 to 23.23)	(−28.87 to −6.20)	(−50.24 to −25.38)	(−16.16 to 12.65)
Stationary cart share	−4.60	−5.50**	−3.59	−6.05**	−3.24	−0.38	−4.30
	(−9.43 to 0.22)	(−9.53 to −1.47)	(−9.98 to 2.79)	(−9.46 to −2.65)	(−10.66 to 4.17)	(−7.78 to 7.03)	(−9.39 to 0.78)
Observations	12,016	4,839	7,177	5,996	6,020	3,536	8,480
**B. High blood glucose (%)**	**Total**	**Male**	**Female**	**Low income**	**High income**	**Sri Lanka**	**Bangladesh**
FFR share	0.05	0.03	0.09*	0.07	0.03	−0.02	0.05
	(−0.01 to 0.11)	(−0.05 to 0.10)	(0.01 to 0.16)	(−0.02 to 0.16)	(−0.04 to 0.10)	(−0.08 to 0.04)	(−0.02 to 0.12)
Supermarket share	−0.08	0.14	−0.39**	−0.13	−0.04	−0.01	−0.42**
	(−0.26 to 0.10)	(−0.08 to 0.35)	(−0.68 to −0.10)	(−0.36 to 0.10)	(−0.24 to 0.17)	(−0.19 to 0.18)	(−0.66 to −0.18)
Corner store share	−0.01	−0.02	−0.00	0.01	−0.03	−0.03	−0.00
	(−0.04 to 0.02)	(−0.05 to 0.02)	(−0.03 to 0.03)	(−0.01 to 0.03)	(−0.08 to 0.01)	(−0.07 to 0.02)	(−0.04 to 0.03)
Mobile cart share	−0.13	−0.01	−0.24**	−0.06	−0.20**	−0.38**	−0.07
	(−0.28 to 0.02)	(−0.13 to 0.12)	(−0.39 to −0.08)	(−0.29 to 0.17)	(−0.33 to −0.08)	(−0.58 to −0.18)	(−0.18 to 0.03)
Stationary cart share	−0.06	−0.07*	−0.05	−0.08*	−0.05	0.01	−0.06
	(−0.14 to 0.01)	(−0.13 to −0.01)	(−0.15 to 0.04)	(−0.14 to −0.02)	(−0.14 to 0.03)	(−0.05 to 0.07)	(−0.13 to 0.01)
Observations	12,003	4,827	7,169	5,992	6,003	3,523	8,480
**C. Diagnosed DM (%)**	**Total**	**Male**	**Female**	**Low income**	**High income**	**Sri Lanka**	**Bangladesh**
FFR share	0.08*	0.06*	0.10*	0.07*	0.09*	0.02	0.02
	(0.02 to 0.14)	(0.00 to 0.12)	(0.01 to 0.18)	(0.00 to 0.13)	(0.01 to 0.17)	(−0.06 to 0.09)	(−0.06 to 0.09)
Supermarket share	0.06	0.08	0.03	0.09	0.05	0.15**	0.15**
	(−0.06 to 0.17)	(−0.09 to 0.24)	(−0.19 to 0.25)	(−0.06 to 0.23)	(−0.12 to 0.23)	(0.07 to 0.24)	(0.07 to 0.24)
Corner store share	0.01	−0.00	0.01	0.01	−0.01	−0.01	−0.01
	(−0.01 to 0.02)	(−0.02 to 0.02)	(−0.01 to 0.04)	(−0.01 to 0.04)	(−0.03 to 0.02)	(−0.04 to 0.01)	(−0.04 to 0.01)
Mobile cart share	−0.06	0.03	−0.16	−0.03	−0.12	−0.19	−0.19
	(−0.21 to 0.09)	(−0.05 to 0.12)	(−0.38 to 0.06)	(−0.23 to 0.17)	(−0.27 to 0.04)	(−0.51 to 0.13)	(−0.51 to 0.13)
Stationary cart share	−0.02	0.00	−0.04	−0.04	−0.01	−0.02	−0.02
	(−0.08 to 0.04)	(−0.06 to 0.06)	(−0.11 to 0.02)	(−0.10 to 0.02)	(−0.09 to 0.06)	(−0.08 to 0.05)	(−0.08 to 0.05)
Observations	12,079	4,854	7,217	6,027	6,038	3,573	3,573

Note: The values show OLS regression coefficients in panel A and AMEs from logistic regressions in panels B and C; 95% CIs in brackets. Density/share of outlets = the number of each outlet out of the total number of outlets. For example, supermarket share is defined as the number of supermarkets within a 300-m buffer around participant’s home address out of all food outlets within a 300-m buffer. Control variables included demographics, SES, health status, healthcare utilization, and physical activities—see Table 1, panel A. All regressions include site fixed effects to control for site-specific time invariant confounders. Level of significance =

* *p* < 0.05

** *p* < 0.01.

AME, average marginal effect; DM, diabetes mellitus; FFR, fast-food restaurant; OLS, ordinary least squares; SES, socioeconomic status.

**Table 3:** Associations between food outlet proximity and fasting blood glucose (OLS coefficients), high blood glucose, and diagnosed DM (AORs).

**A. Blood glucose level**	**Total**	**Male**	**Female**	**Low income**	**High income**	**Sri Lanka**	**Bangladesh**
FFR proximity	2.14**	3.15*	1.36	0.36	3.84*	2.45	1.64
	(0.55 to 3.72)	(0.39 to 5.91)	(−0.38 to 3.11)	(−2.32 to 3.03)	(0.68 to 7.00)	(−0.83 to 5.73)	(−0.71 to 3.99)
Supermarket proximity	−0.72	1.14	−1.20	0.46	−1.35	−0.45	−0.53
	(−2.48 to 1.04)	(−5.45 to 7.73)	(−3.74 to 1.34)	(−3.94 to 4.85)	(−3.08 to 0.38)	(−4.27 to 3.37)	(−2.16 to 1.09)
Corner store proximity	1.32	0.19	2.08	1.83	0.82	−2.34	2.58
	(−1.69 to 4.33)	(−2.99 to 3.36)	(−1.55 to 5.71)	(−1.31 to 4.98)	(−2.39 to 4.04)	(−6.09 to 1.42)	(−1.27 to 6.43)
Mobile cart proximity	1.51	0.19	2.25	2.92	0.60	−0.17	2.69
	(−1.73 to 4.74)	(−4.79 to 5.18)	(−2.52 to 7.03)	(−2.39 to 8.24)	(−3.54 to 4.74)	(−4.15 to 3.81)	(−1.40 to 6.77)
Stationary cart proximity	0.58	0.25	0.65	1.51	−0.53	3.60	−0.32
	(−2.43 to 3.60)	(−3.04 to 3.54)	(−2.60 to 3.91)	(−2.17 to 5.19)	(−4.26 to 3.20)	(−0.38 to 7.57)	(−4.45 to 3.82)
Observations	12,016	4,839	7,177	5,996	6,020	3,536	8,480
**B. High blood glucose (%)**	**Total**	**Male**	**Female**	**Low income**	**High income**	**Sri Lanka**	**Bangladesh**
FFR proximity	1.16*	1.29*	1.08	0.98	1.37**	0.92	1.23*
	(1.01 to 1.33)	(1.03 to 1.63)	(0.88 to 1.33)	(0.65 to 1.48)	(1.13 to 1.67)	(0.77 to 1.10)	(1.03 to 1.47)
Supermarket proximity	1.00	1.18	0.95	0.95	0.99	0.98	1.18**
	(0.86 to 1.17)	(0.87 to 1.61)	(0.66 to 1.36)	(0.50 to 1.80)	(0.72 to 1.37)	(0.79 to 1.21)	(1.09 to 1.27)
Corner store proximity	1.08	1.06	1.12	1.26	0.95	0.85	1.22
	(0.87 to 1.35)	(0.80 to 1.41)	(0.87 to 1.42)	(0.96 to 1.65)	(0.71 to 1.26)	(0.68 to 1.06)	(0.93 to 1.60)
Mobile cart proximity	0.99	0.92	1.05	0.99	0.96	0.96	1.02
	(0.75 to 1.31)	(0.68 to 1.23)	(0.74 to 1.48)	(0.59 to 1.67)	(0.75 to 1.24)	(0.70 to 1.32)	(0.71 to 1.48)
Stationary cart proximity	1.15	1.12	1.13	1.12	1.14	1.34*	1.06
	(0.94 to 1.40)	(0.78 to 1.61)	(0.90 to 1.43)	(0.84 to 1.49)	(0.92 to 1.40)	(1.07 to 1.68)	(0.79 to 1.43)
Observations	12,003	4,827	7,169	5,992	6,003	3,523	8,480
**C. Diagnosed DM (%)**	**Total**	**Male**	**Female**	**Low income**	**High income**	**Sri Lanka**	**Bangladesh**
FFR proximity	1.19*	1.22	1.16	1.09	1.33**	1.08	1.27**
	(1.03 to 1.38)	(0.90 to 1.65)	(0.96 to 1.40)	(0.81 to 1.45)	(1.08 to 1.64)	(0.75 to 1.55)	(1.09 to 1.49)
Supermarket proximity	1.09	1.36*	0.99	0.80	1.20	1.00	1.40**
	(0.79 to 1.51)	(1.07 to 1.73)	(0.64 to 1.52)	(0.45 to 1.41)	(0.83 to 1.74)	(0.61 to 1.63)	(1.27 to 1.55)
Corner store proximity	1.12	1.10	1.16	1.29*	0.98	0.90	1.27
	(0.92 to 1.37)	(0.82 to 1.47)	(0.88 to 1.52)	(1.05 to 1.59)	(0.73 to 1.33)	(0.69 to 1.18)	(0.98 to 1.66)
Mobile cart proximity	0.99	0.80	1.08	1.01	0.94	1.24*	0.89
	(0.77 to 1.26)	(0.58 to 1.10)	(0.71 to 1.64)	(0.67 to 1.53)	(0.74 to 1.19)	(1.00 to 1.54)	(0.66 to 1.19)
Stationary cart proximity	1.25*	1.27	1.18	1.24	1.26	1.07	1.30
	(1.00 to 1.56)	(0.91 to 1.78)	(0.86 to 1.62)	(0.87 to 1.78)	(0.93 to 1.71)	(0.78 to 1.48)	(0.99 to 1.70)
Observations	12,079	4,854	7,217	6,027	6,038	3,573	8,506

Note: The values show OLS regression coefficients in panel A and AORs from logistic regressions in panels B and C; 95% CIs in brackets. Proximity of outlets = 1 if at least 1 FFR within 100 m and 0 if otherwise. Control variables included demographics, SES, health status, healthcare utilization, and physical activities—see Table 1, panel A. All regressions include site fixed effects, where in all regressions we controlled for site-specific time invariant characteristics. Level of significance =

* *p* < 0.05

** *p* < 0.01.

AOR, adjusted odds ratio; DM, diabetes mellitus; FFR, fast-food restaurant; OLS, ordinary least squares; SES, socioeconomic status.

**Fig 2:**
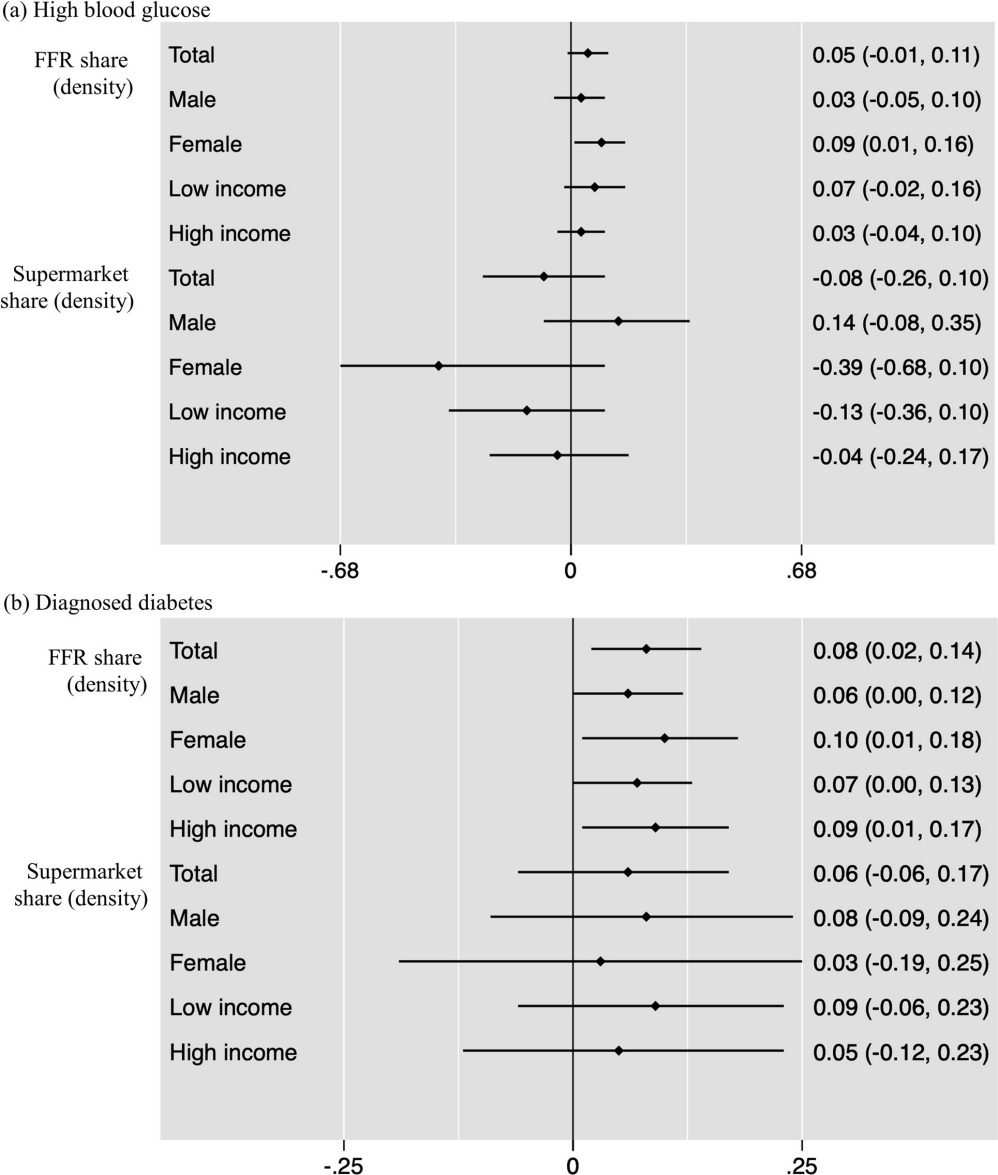
AMEs for the associations between diabetes mellitus and density food outlets. Note: The values show AME from logistic regressions in panels B and C; 95% CIs in brackets; horizontal bars = 95% CIs. Density = the number of each food outlet per total number of food outlets within 300 m of a resident’s home address. Proximity = 1 if having at least 1 outlet within 100 m of a resident’s home address and 0 if otherwise. AME, average marginal effect; FFR, fast-food restaurant.

**Fig 2** shows the associations between the density of food outlets and diabetes mellitus (**Tables 2 and 3**). Overall, no statistically significant results were found for the share of supermarket, corner stores, stationary carts, and mobile carts (**Table 2,** panels B and C). However, results showed that the density/share of FFR in the neighborhood of individuals’ homes was positively associated with the probability of being diagnosed with diabetes. A 1% increase in the share of FFR near an individual’s home was associated with 8% increase in the probability of being clinically diagnosed as a diabetic (AME: 0.08; CI: 0.02, 0.14; *p* < 0.05). When stratifying by sex, while stronger among females (AME: 0.10; CI: 0.01, 0.18; *p* < 0.05) and higher-income populations (AME: 0.09; CI: 0.01, 0.17; *p* < 0.05), these associations were also statistically significant in males (AME: 0.06; CI: 0.001, 0.12; *p* < 0.05) and lower-income populations (AME: 0.07; CI: 0.001, 0.13; *p* < 0.05).

**Fig 3:**
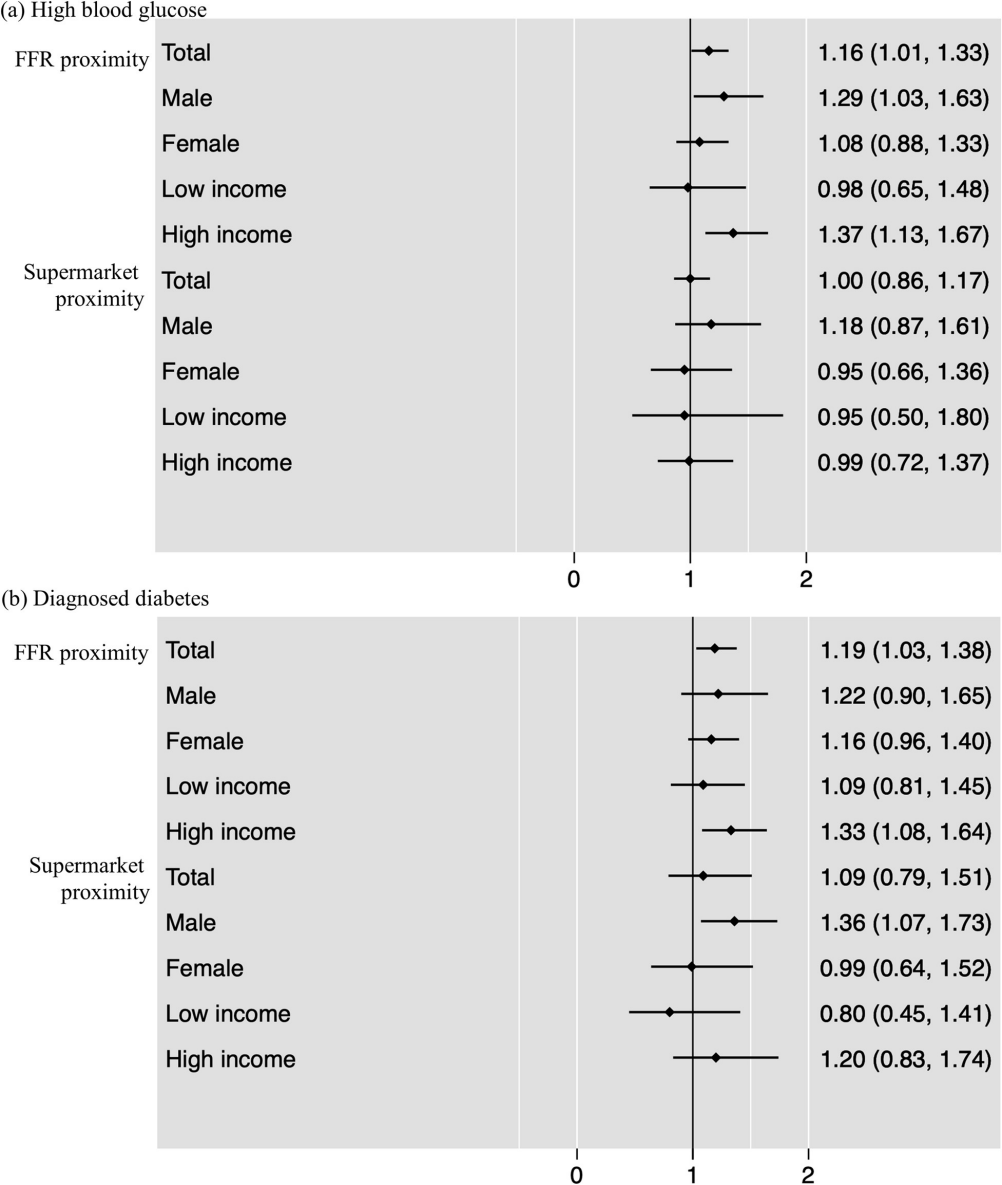
ORs for the associations between diabetes mellitus and proximity of food outlets. Note: The values show AORs from logistic regressions in panels B and C; 95% CIs in brackets; horizontal bars = 95% CIs. Density = the number of each food outlet per total number of food outlets within 300 m of a resident’s home address. Proximity = 1 if having at least 1 outlet within 100 m of a resident’s home address and 0 if otherwise. AOR, adjusted odds ratio; FFR, fast-food restaurant; OR, odds ratio.

Regarding the associations between the proximity of food outlets and diabetes mellitus, no statistically significant results were found for the share of supermarket, corner stores, stationary carts, and mobile carts (**Table 3**, panels B and C). However, results showed that the proximity of FFR near home was positively associated with the probability of having high blood glucose level and being diagnosed with diabetes. Having at least 1 FFR near home was associated 16% (odds ratio [OR]: 1.16; CI: 1.01, 1.33; *p* < 0.05) and 19% (OR: 1.19; CI: 1.03, 1.38; *p* < 0.05) increases in the odds of having high blood glucose and being diagnosed diabetes, respectively. The association between FFR proximity and having high blood glucose was statistically significant for males (OR: 1.29; CI: 1.03, 1.63; *p* < 0.05) but not females, although the effect sizes are of similar order of magnitude. Associations of the proximity to FFR and high blood glucose were statistically significant only for high-income earners (OR: 1.37; CI: 1.13, 1.67; *p* < 0.01). The association between FFR proximity and having a diabetes mellitus diagnosis was statistically significant only in those with higher income (OR: 1.33; CI: 1.08, 1.64; *p* < 0.01) (**Fig 3**, **Tables 2 and 3**).

## Discussion

We examined the associations of density and proximity of healthy and unhealthy food outlets with T2DM, as well as the heterogeneity of such associations by sex and income in Bangladesh and Sri Lanka. We used a unique dataset that merged individual-level surveillance data (South Asia Biobank) with built environment data measuring individual exposure to healthy and unhealthy food outlets.

We found that the share of FFR was higher in Bangladesh (among the country with the biggest number of people with T2DM in 2019) [1], while the shares of corner stores and supermarkets were higher in Sri Lanka. All supermarkets were identified in urban sites. We identified that T2DM levels were higher among females and higher-income participants.

When testing the association of the food environment and diabetes mellitus, no significant findings were identified in relation to the share of supermarket, corner stores, stationary carts, nor mobile carts. However, an observed key finding of our study was that FFR share, density, and proximity were all associated with a greater risk of T2DM. These findings are consistent with previous evidence from high-income countries such as UK and Sweden where unhealthy food outlets have been found to increase the odds of developing T2DM [4,5].

With regard to sex differences, a higher FFR density was associated with greater blood glucose, higher likelihood of high blood glucose for female but not male, and higher chances of being diagnosed with DM for both female and male. Proximity to FFR was associated with a higher blood glucose level in males but does not seem to play a role in the likelihood of being diagnosed with diabetes for both male and female. These sex differences could be due to differences in exposure metrics—density and proximity—capturing different dimensions of decision-making for food sourcing. Previous studies have shown differences in how men and women engage differently to the food environment [4]. Therefore, our finding could be explained by how sex affects perception of and interaction with the surrounding environment. With lower labor market participation than men in these countries, women spend more time with household chores including sourcing foods, and, therefore, it is plausible that they get more exposed to a range of outlets around their homes [4], rendering density of outlets more salient in their food choices. Labor market participation of women in Sri Lanka is particularly low in urban environments [22] that tend to be characterized by wider availability of FFRs. With higher labor market participation, men are more time constrained due to employment and thus interact with the environment on more sporadic contexts where proximity and convenience become relevant factors for decision-making [4].

Another possible channel for these associations may be sex differences with regard to overall dietary preferences and habitual dietary intakes. For example, previous studies have shown that women have a higher intake of sugar compared with men. To further unpack these associations, one would need time use survey combined with a mapping of how each individual interacts with the environment (e.g., where they shop, how frequently, and what they buy).

While our data do not enable us to assess these mechanisms, the sex differences on the role of the food environment on diabetes have important public health implications. Because South Asians have a greater visceral adiposity and insulin resistance, impaired β-cell function, and a genetic predisposition to diabetes, which culminates in a markedly increased risk that may lead to the development of T2DM [23], they should be targeted for prevention. South Asian women in particular are more likely to have diabetes than white women [24,25]. While the diagnosis of diabetes doubles the cardiovascular risk in men, it more than triples the risk in women. Women are also at greater risk of other diabetes-related complications such as blindness, kidney disease, and depression [24]. Therefore, our results suggest that interventions in the food environments may be particularly important for women in these countries. Examples of interventions include the subsidization of healthy food and taxation of fast food, nutritional labeling in menus in FFRs, and nutritional literacy interventions that discourage the consumption of fast food.

Our findings also showed that T2DM risk variations by income, with exposure (through density and proximity) to FFRs affecting more blood glucose and likelihood of being diagnosed diabetes for high-income earners. These findings are consistent with existing evidence that shows diabetes being more prevalent among the wealthy groups [26]. With rapid shifts in lifestyle characterized by a nutritional transition and urbanization [27], the increased availability and accessibility of unhealthy food, combined with a raise in economic purchasing power, generates greater opportunities to eat out of home, which, with the proliferation of fast-food establishments, may contribute to the formation of unhealthy habitual dietary patterns [28]. In LMICs, western shops selling processed foods and fast-food restaurants strategically locate in higher-income neighborhoods [29] to target those that can afford these foods. These foods also tend to be more expensive than fresh produce and FV. Therefore, these could explain why upper-income populations are more affected by being exposed to more obesogenic environments.

In addition, evidence also suggests that the wealthier tend to shop at conveniently located stores around their neighborhoods, while low-income individuals are more likely to travel long distances to shop at cheaper markets or street stalls [30]. Therefore, it is plausible that exposure to unhealthy environments plays a stronger role in explaining prevalence of diabetes for the wealthier than for those with low income.

These findings highlight the need for public health interventions targeting high-income earners. Given that affordability may not be a key factor for decision-making among high-income earners, diabetes prevention strategies should include improving the saliency and convenience of accessing healthy foods, as well as improving nutritional literacy.

However, preventive strategies are also required to prevent lower-income populations to develop risk factors that may lead T2DM. Indeed, although to a lesser extent than for high-income earners, our results suggest that density of fast foods is positively associated with the likelihood of being diagnosed with diabetes also for low-income earners. Recent evidence on obesity trends, a risk factor of diabetes, shows that as the LMIC’s gross domestic product (GDP) increases the rates of obesity among low SES group increases with the shift of obesity occurring first in low SES women [31]. Due to financial, educational, mobility, and time constrains, low-income populations may have less opportunities to consume healthy foods [32]. Thus, food environments that are more prone to facilitate unhealthy food choices in combination with the absence of fiscal policies and regulations on access to healthy foods may affect more disproportionally the lower-income groups in the future to come. Therefore, upstream policies guided by a ‘health in all policies’ approach that explicitly target the key social, economic, and structural determinants of health and behaviors may become essential to prevent diabetes [33]. Such approaches may include unhealthy food and beverage taxation, subsidies for healthy foods, food labeling on menus, banning unhealthy food advertising, as well as promoting better urban planning and subsidized transport that facilitate access to healthy foods and encourage physical activity [33].

To summarize, consistent with other research in South Asia [11], our results suggest interventions targeting the environment may be effective in preventing diabetes; however, the heterogeneity of the associations found in our analysis suggests that more specific interventions may be needed. This is aligned with other evidence indicating that one-size-fits-all built environment interventions have not led to improved outcomes [12], and future research is needed to evaluate which food environment interventions could improve diabetes outcomes in this geographical region and population.

There are at least 5 limitations of our study. First, since we used cross-sectional individual level data, temporality in the associations cannot be established. Second, given the cross-sectional nature of the data, our study is descriptive and does not enable identifying the causal impacts of the environment on the outcomes assessed. While we control for a range of cofounders, we cannot fully address all endogeneity and reverse causality concerns (e.g., unobserved food preferences of those at risk of diabetes may be such that they locate in places with a high density of unhealthy food outlets). Third, we captured only part of the environment with the 300-m buffer. Even though this is commonly used in the literature to examine built environment and health behavior/outcomes [18,19], this approach implies that we may imperfectly capture exposure to obesogenic food environments as we do not measure whether participations shop beyond the assessed areas. To investigate those effects in a meaningful way would require an understanding of where individuals gravitate beyond their residential neighborhoods to infer their individual level exposure to different food environments. Despite this limitation, focusing on a narrow definition of the built environments around homes has the advantage of considering outlets in areas where individuals are highly likely to gravitate to during the day and are, therefore, likely to use. Importantly, some of the drawbacks of considering small buffers in countries where food outlets are clustered in different parts of towns (e.g. large supermarkets outside of the city center, while fast food outlets in commercial and business areas), are mitigated in the geographies we assess, where there is an abundance of a variety of food outlets in the immediacies of individual’s homes. Fourth, we measured food environments using residency geolocation; however, participants may consume part of their meals far from home (e.g., if the workplace is distant). Also, we measured exposure based on residency rather than relying on individual shopping data and consumption patterns. Such data are not available, although it could be important to further identify the heterogeneity of the observed associations. Fifth, we categorized the extent to which food environments are healthy and unhealthy based on the international classification in the absence of a classification for South Asian countries. Since we did not observe food sold in these food outlets and lacked consumption data, the magnitude of the associations may reflect the mix of healthy and unhealthy foods available in these outlets in particular supermarkets. Despite these caveats, our study provides novel evidence on the association between food environment and T2DM as well as its unequal associations by sex and income.

## Conclusions

To our knowledge, this is the first study to assess the association of the food environment and T2DM in LMICs by merging health outcome data and food outlet geolocations. The accessibility and availability of fast-food restaurants pose a greater risk for the development of T2DM in South Asia. Considering the complex interplay between food outlets and their in-store food environment, it is important to understand the mechanisms and confirm the causal implications of these findings. Policy actions are required to improve the quality of food environments in South Asian countries and other LMICs for the prevention of NCDs such as T2DM.

## Disclaimers

The views expressed in this publication are those of the author(s) and not necessarily those of the NIHR, the UK Department of Health and Social Care, or the ESRC.

## Supporting information

S1 Fig Sample images of mobile food carts from environmental mapping. (DOCX)

S1 Text Model specifications and CONSORT diagram. CONSORT, Consolidated Standards of Reporting Trials.
(DOCX)

S2 Text Planned data collection, outcome variables, and data analyses. (DOCX)

S1 Table Variables definition. (DOCX)

S2 Table Unadjusted regressions. (DOCX)

S3 Table STROBE Statement for cross-sectional studies. STROBE, Strengthening the Reporting of Observational Studies in Epidemiology.
(DOCX)

## Abbreviations

AME: average marginal effect
AOR: adjusted odds ratio
FFR: fast-food restaurant
FV: fruits and vegetables
GDP: gross domestic product
GHRU: Global Health Research Unit
GIS: geographic information system
LMIC: low- and middle-income country
NCD: noncommunicable disease
OLS: ordinary least squares
OR: odds ratio
PPP: purchasing power parity
SES: socioeconomic status
STROBE: Strengthening the Reporting of Observational Studies in Epidemiology
T2DM: type 2 diabetes mellitus
