# Supplementary figures and images for: Food environment and diabetes mellitus in South Asia: A geospatial analysis of health outcome data
Source: PLoS Med. 2022 Apr 26;19(4):e1003970. doi: 10.1371/journal.pmed.1003970 (PMC9041866; doi:10.1371/journal.pmed.1003970)

**Sample images of mobile food carts from environmental mapping**


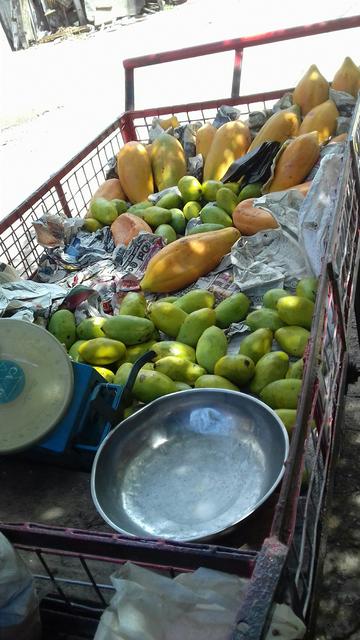

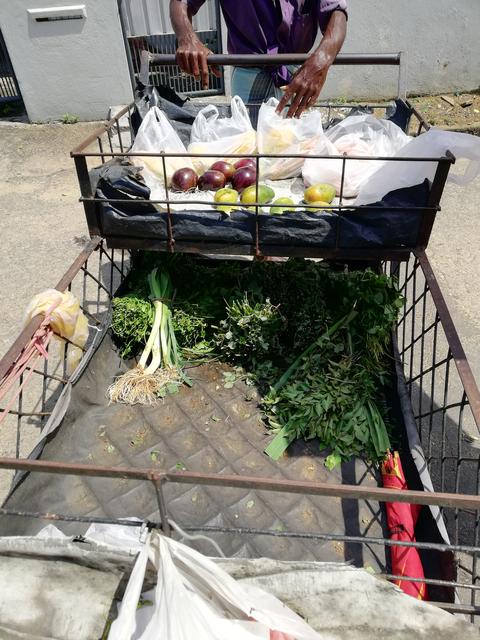


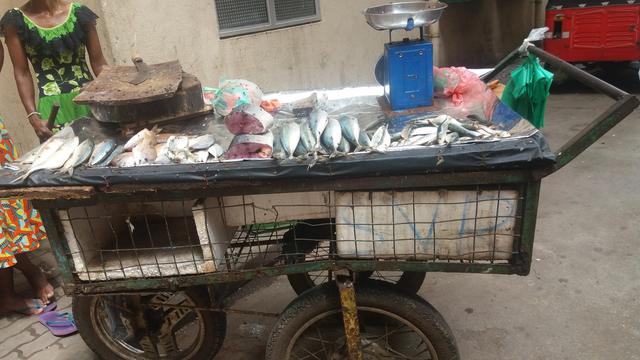


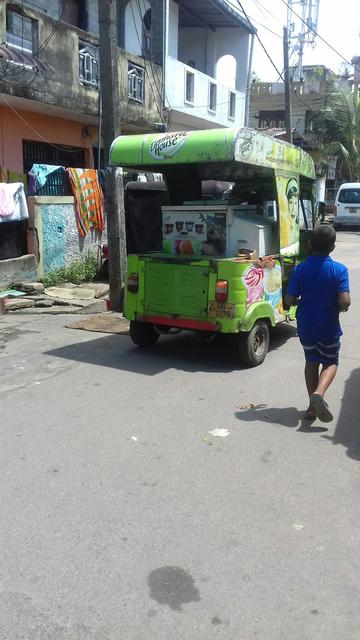

Supplement: S1 Fig — (DOCX) [file pmed.1003970.s001.docx]
