# Supplementary material for: Food environment and diabetes mellitus in South Asia: A geospatial analysis of health outcome data
Source: PLoS Med. 2022 Apr 26;19(4):e1003970. doi: 10.1371/journal.pmed.1003970 (PMC9041866; doi:10.1371/journal.pmed.1003970)
Supplement: S1 Text — CONSORT, Consolidated Standards of Reporting Trials. (DOCX) [file pmed.1003970.s002.docx]

**Model specifications & CONSORT diagram**

**A. Model specifications**

For the glucose variable we ran a linear regression analysis using OLS site fixed effects specification:

$Y_{ij}=\beta_{0}+ \beta_{1}{Outlet\_{Share}_{ij}^{k}}+\beta_{2}X_{ij}+ \psi_{j}+ \mathcal{E}_{ij}$ (1)

Whereby $Y_{ij}$ is the glucose for individual $i$ in site $j$*. Outlet*$\_{Share}_{ij}^{k}$ is the number of each outlet (FFR, SP, ST, MB, CR) out of all outlets within a radius of $k=300 meters.$ Previous work indicates that individuals respond differently to food exposure based on the area of living and their sex (Dunn et al., 2010, Currie et al., 2010). Therefore, separate regressions were ran by splitting the sample by sex and income.

For the outcome variable *diabetes/diagnosed diabetes* we ran a logit using the following model:

$\Pr\left( {DM}_{ij}=1 \right)=F\left( X \right)=F( \beta_{0}+ \beta_{1}{Outlet\_{Share}_{ij}^{k}}+\beta_{2}X_{ij}+ \psi+ \mathcal{E}_{ij} )$ (2)

Where F(.) is the logistic function, where $\Pr\left( {Diabetes}_{ij}=1 \right)$ is the probability of the individual $i$ in site $j$ to have glucose 126+, as a function of the key variables of interest, *Outlet*$\_{Share}_{ij}^{k}$ that measure the number of each type of outlet out of all outlets within a radius of *k = 300* meters from the residency of individual $i$ in site $j$. $X$, -$\psi$, and $\mathcal{E}_{ij}$ are the same as defined above. Similar regressions for proximity.

**B. CONSORT diagram on sample selection process**

Observations with health and geolocation data:
N=12167 (3633 SL; 8534 BD)

Observations with missing or implausible values:

N=104 for socio-economic, demographic, and self-assessed health variables

N= 3494 for healthcare utilization variables

Observations:

N= 12063 Glucose level

N= 12063 Raised glucose

N= 12079 Diagnosed diabetes

Total observations analysed per outcome variable:

N= 12016 Glucose level

N= 12016 Raised glucose

N= 12079 Diagnosed diabetes

*Note: Several observations had missing values namely: n=104 had missing socio economic and self-assessed health characteristics missing (n=14 observations had sex missing; n=88 observations had missing Employment Status, Marital Status, School Years, Monthly Income, Number of Household Residents, Self Assessed Health; n=2 with implausible age values (e.g. age reported as 5233) and age under 18 and were, therefore, set to missing), n= 3494 observations had missing values for – Advice on losing weight; Advice on increase fruit and vegetable consumption; Advice on reducing fat consumption; Advice on reducing sugar consumption.*

**References**

Currie J, DellaVigna S, Moretti E, Pathania V. The effect of fast food restaurants on obesity and weight gain. American Economic Journal: Economic Policy. 2010 Aug;2(3):32-63.

Dunn RA. The effect of fast‐food availability on obesity: an analysis by gender, race, and residential location. American Journal of Agricultural Economics. 2010 Jul;92(4):1149-64.

Song P, Gupta A, Goon IY, Hasan M, Mahmood S, Pradeepa R. Data Resource Profile: Understanding the patterns and determinants of health in South Asians—the South Asia Biobank. Int J Epidemiol 2021;50:717-718e.
