# Supplementary material for: Food environment and diabetes mellitus in South Asia: A geospatial analysis of health outcome data
Source: PLoS Med. 2022 Apr 26;19(4):e1003970. doi: 10.1371/journal.pmed.1003970 (PMC9041866; doi:10.1371/journal.pmed.1003970)
Supplement: S2 Text — (DOCX) [file pmed.1003970.s003.docx]

**Planned data collection, outcome variables, and data analyses**

This document contains excerpts of the research proposal submitted to the funder where we explain the items relevant for the research presented in this paper as well as the research protocol. We first present the planned data collection as explained (section A) and then present the planned analyses that is the focus of this paper (section B).

**A. Data Collection and outcome variables**

**Surveillance study design**

We will establish a network of community based, NCD surveillance sites in Bangladesh, India North, India South, Pakistan and Sri Lanka (N~40 per geographic region; N~200 in total). Surveillance sites will be centred on primary, community healthcare units, and distributed in representative rural and urban settings. At each of the ~200 surveillance sites, we will carry out systematic, structured assessments of ~750 people from the local, resident adult population (age 18+ yrs), to quantify i. the burden of CVD and T2D, and their risk factors, and ii. the quality of preventative care and disease management for CVD and T2D in the population. Data collection is specifically designed to provide quantitative data on the core NCD surveillance indicators in the WHO Global Monitoring Framework.

Data will be collected using shared methodology and standardised operating procedures, and will include measurement of blood based biomarkers for T2D and CVD. Results will be captured electronically to provide a rich and robust resource on ~160,000 men and women from across South Asia, and made available to the health policy makers and other stakeholders to enable assessment of the needs and priorities of the population for NCD prevention and control. In addition biological samples (whole blood, serum and urine) will be collected from the 160,000 people screened to provide a unique and powerful resource for high-quality molecular epidemiological research into the mechanisms underlying, and the biomarkers predicting, the high rates of T2D and CVD in South Asians.

**Recruitment**

At each study site, governmental census data and other available household listings will used, supported by house-house visits, to fully enumerate and identify the resident population. House-to-house visits will be done by the local primary care worker, in partnership with the research team. The purpose and potential benefits (personal and societal) of the surveillance will be explained. The research team will then obtain demographic details for the adult population of each household. People eligible and consenting to take part in the surveillance study will be asked to attend a nearby health facility in the fasting state, for the surveillance health assessment. Explanations of the project will be provided in writing and using videos, available in relevant South Asian languages, and supported by bilingual translators. We will also engage the community elders (eg teachers, employers, religious leaders) as trusted third parties, to support and facilitate engagement of the community in the study. Based on previous experience with population studies in these communities, participation rates are expected to exceed >70%; the availability of household enumeration data will enable participation to be monitored in detail, including by age, gender and major socio-economic groups.

**Study procedures**

Participants will be asked to attend in the morning after an overnight fast (water only after midnight). All participants will complete a structured assessment by trained members the dedicated research team, in five complementary domains: i. Registration and consent; ii. Questionnaire; iii. Physical measurements; iv. Biological samples and v. Clinical reporting. All study procedures implemented represent well established tools, that are validated for assessment of NCDs in population studies. The procedures will be carried out by trained research staff, using equipment and protocols that are standardized between countries and surveillance sites.

Registration and consent. Written, informed consent will be obtained from all participants for data collection, and inclusion in the research. Informed consent will include permission for the data and samples collected to be used for NCD research, including data sharing with national and international bodies concerned with prevention and control of T2D and CVD, as well as for molecular epidemiological research. Consent will be facilitated using videos (available in major South Asian languages) and supported by bilingual translators. A unique study ID will be allocated to each participant.

Questionnaire. An interviewer administered health and lifestyle questionnaire will be used to collect information on behavioral risk factors (smoking, alcohol habit, physical activity and fruits/vegetables consumption), personal and family medical history, medications, socio-economic status and knowledge of NCDs. The questionnaire is founded on the extended WHO STEPS questionnaire that is widely used in global NCD surveillance,17 but adapted for use in South Asia, including through incorporation of additional questions. The interviewer administered questionnaires will be translated into locally relevant languages, and supported by graphics representations to facilitate collection of high quality data.

Physical measurements. Including: a) Anthropometry (height, weight, waist and hip circumference, and bio-impedance for body fat composition); b) Blood pressure by digital device; c) Cardiac evaluation by 12 lead ECG to identify arrhythmia, LVH and previous myocardial infarction; d) Retinal photography for assessment of retinal disease, including hypertensive and diabetic retinopathy; and e). Respiratory evaluation by spirometry to assess for smoking/environment-related lung injury.

Biological samples. We will collect venous blood samples (~20mls, by venesection using trained phlebotomists) for measurement of fasting glucose, lipid profile and HbA1c and other clinical relevant markers of cardiovascular health. This will comprise collection of blood into EDTA (~8ml), serum (~8ml) and citrate (~4ml) vacutainer tubes. An Oral Glucose Tolerance Test will be carried out in a subset of participants, enabling validation of diabetes classification by HbA1c. A spot urine sample (10ml) will also be collected for analysis of albuminuria and other biomarkers. Laboratory assays will be carried out using validated near-patient assays, and aliquots stored (-80C) for both external QC and future molecular epidemiological research.

Clinical reporting. All results will be reviewed for clinically significant findings by a medically qualified member of the research team, who will also be responsible for ensuring that participants identified to have significant health conditions (e.g. T2D, hypertension) are referred to an appropriate facility for counseling and treatment. All study participants will receive a clinical report detailing the results of their health assessment, and the opportunity to discuss their results with the medical practitioner if significant abnormalities are present. To facilitate this, the clinical report will be made available to local community healthcare teams. Results will also be accompanied by a booklet of explanation, as well as access to an explanatory video.

**B. Data analyses**

Workplan

Specific aim 1. Comprehensive assessment of environmental influence on health risky behaviours and health In GHRU2, we will conduct comprehensive analyses by linking the surveillance and environment mapping data. The surveillance data include socioeconomic variables (age, gender, income, education, and employment), food related behaviours, and health outcomes such as CVD (heart attack and stroke), T2D (diagnosed and undiagnosed diabetes), and their main risk factors (overweight and obesity, physical inactivity, tobacco smoking and chewing, alcohol drinking, and raised blood pressure and cholesterol. The environment data include geolocations of food/tobacco retailers (e.g. supermarket, corner stores, public markets, mobile carts, and restaurants), geolocations of physical activity facility (e.g. recreational centre, public parks, walking/cycling paths, sport clubs, playgrounds) and schools, availability of healthy food (e.g. fruits, vegetables) and ‘unhealthy’ food (e.g., confectionary, fried snacks, soft drinks, sweet biscuits), outdoor advertising of food and tobacco, and warning labels of cigarettes and bidis (**Figures 4.2**). The data is linked at individual level using place of residence, so that for each individual we characterize their individual exposure to the built environment. Currently, we have 52 surveillance sites that have completed both the surveillance data collection (26,000+ participants) and environment mapping data collection (estimated 6000+ retailers and 18,000+ images of adverts, facilities, tobacco packages) in Sri Lanka, Bangladesh, South India, and North India. We will leverage these data to: i) develop and validate indicators of built environment exposure; ii) assess the influence of the built environment on health risky behaviour (food choices, nutritional quality, smoking and physical inactivity) and BMI and health outcomes; iii) assess heterogeneity of the effects of the built environment on the main outcomes by socio economic characteristics. Given that our baseline data is pre-pandemic, where feasible, we will also leverage on pandemic related shocks that impacted the built environment to identify the causal impact of the environment on the outcomes of interest.

For i) and ii), the primary research questions will include (a) whether better access to ‘unhealthy’ food outlets is associated with higher BMI; (b) whether better access to ‘healthy’ food outlets (esp. fruit and vegetables) is associated with better diet (e.g. higher consumption of fruit and vegetables, higher diet quality index), and lower and BMI; (c) whether higher density of retailers and adverts of tobacco (cigarette and bidi) is associated with higher tobacco use (smoking and chewing); and (d) whether proximity to physical activity facilities is associated with lower physical inactivity among the community. Our analyses will combine the use of mapping software (e.g. ArcMap, QGIS) and statistical software (e.g. Stata, R). In collaboration with the NIHR Global Health Group on Diet and Activity Research (GDAR) in Cambridge, we will also work with Burgoine, Foley and Woodcock to bring methodological and subject-specific expertise to the collection and analysis of more comprehensive data on the upstream determinants of food intake and domain-specific physical activity behaviour in South Asia. The insights from these findings will be complemented with results from the pilot of Healthy Village Plus to fine tune the scope of the HVP interventions to be scaled up at national level by the HPB (SA2).
