# Supplementary material for: Food environment and diabetes mellitus in South Asia: A geospatial analysis of health outcome data
Source: PLoS Med. 2022 Apr 26;19(4):e1003970. doi: 10.1371/journal.pmed.1003970 (PMC9041866; doi:10.1371/journal.pmed.1003970)
Supplement: S1 Table — (DOCX) [file pmed.1003970.s004.docx]

**Variables Definition**

| **Variable Name** | **Brief definition** |
| --- | --- |
| ***Outcome variables*** |  |
| Blood glucose level | Fasting blood glucose level |
| High blood glucose | Diabetes mellitus, 126+ mg/dl |
| Diagnosed diabetes mellitus | Ever been told by health worker that has raised blood sugar or diabetes or currently taking medications such as insulin |
| ***Main independent Variables*** |  |
| FFR Density (Share) | A continuous variable that measures the share of fast-food restaurants (number of fast-food restaurants out of all food outlets) within 300m from participant address |
| Supermarket Density (Share) | A continuous variable that measures the share of supermarkets (number of supermarkets out of all food outlets) within 300m from participant address |
| FFR Proximity | A binary variable with 1 if at least one fast food restaurant within 100 m and 0 if otherwise |
| Supermarket Proximity | A binary variable with 1 if at least one supermarket within 100 m and 0 if otherwise |
| ***Control Variables*** |  |
| Sex | Binary variable, 1 if female, 0 if male |
| Country | Binary variable, 1 if Bangladesh, 0 if Sri Lanka |
| Age | Continuous variable |
| Marital Status | Binary variable, 1 if married, 0 otherwise |
| Religion | Categorical variable, 1=Buddhist, 2=Christian, 3=Hindu, 4=Muslim, 5=0ther |
| School Years | A continuous variable of number of years in school |
| School Years Squared | Continuous variable, the square term of the variable School Years |
| Income (USD PPP) | Continuous variable capturing the average earning of household per month adjusted using the purchasing power parity rate for 2018. |
| Paid Employment | Binary variable, 1 if on paid employment, 0 otherwise |
| Household Composition | Continuous variable capturing how many people 18+ years old live in the same household |
| Self-Assessed Health | Ordered Categorical variable, 1=poor health, 2=fair,3=good,4=very good, 5=excellent |
| Advice Fruit Veg | Binary variable, 1= received doctor's advice to eat at least five servings of FV a day, 0 otherwise |
| Advice Fat | Binary variable, 1= received doctor's advice to reduce the fat in the diet, 0 otherwise |
| Advice Activity | Binary variable, 1= received doctor's advice to start or do more physical activity, 0 otherwise |
| Advice Weight | Binary variable, 1= received doctor's advice to lose weight, 0 otherwise |
| Advice Sugar | Binary variable, 1= received doctor's advice to reduce sugary beverages intake, 0 otherwise |
| PA Vigorous Work Sport Home Week (mins) | Continuous variable capturing the minutes of vigorous physical activity per week at work, home, or recreational centres; values bigger than 10080 were set as missing (max minutes in a week) (1 value set to missing) |
| PA Moderate Work Sport Home Week (mins) | Continuous variable capturing the minutes of moderate physical activity per week at work, home, or recreational centres; |
| PA Transport Week (mins) | Continuous variable capturing the minutes spent walking or bicycling as a mode of transportation per week |
