# Supplementary material for: Food environment and diabetes mellitus in South Asia: A geospatial analysis of health outcome data
Source: PLoS Med. 2022 Apr 26;19(4):e1003970. doi: 10.1371/journal.pmed.1003970 (PMC9041866; doi:10.1371/journal.pmed.1003970)
Supplement: S2 Table — (DOCX) [file pmed.1003970.s005.docx]

**UNADJUSTED regressions**

**Table A1. UNADJUSTED Associations between food outlet DENSITY and fasting blood glucose (OLS coefficients), high blood glucose and diagnosed DM (average marginal effects)**

| **A. Blood glucose level** | **Total** | **Male** | | **Female** | | **Low income** | | **High income** | | **Sri Lanka** | **Bangladesh** |
| --- | --- | --- | --- | --- | --- | --- | --- | --- | --- | --- | --- |
| FFR share | 10.13 | 5.46 | | 13.45 | | 9.16 | | 8.50 | | -5.17 | 13.23 |
|  | (-1.66 - 21.91) | (-5.17 - 16.10) | | (-1.72 - 28.62) | | (-5.51 - 23.82) | | (-1.82 - 18.82) | | (-18.03 - 7.69) | (-1.70 - 28.16) |
| Supermarket share | -7.71 | 16.61 | | -23.24* | | -17.82 | | 2.30 | | 1.70 | -47.76** |
|  | (-28.46 - 13.04) | (-38.34 - 71.56) | | (-43.34 - -3.14) | | (-38.31 - 2.67) | | (-20.20 - 24.80) | | (-21.13 - 24.53) | (-61.75 - -33.77) |
| Corner store share | -0.24 | -3.12 | | 1.73 | | 1.70 | | -2.05 | | -1.93 | 0.17 |
|  | (-4.53 - 4.05) | (-7.75 - 1.51) | | (-2.98 - 6.43) | | (-2.19 - 5.59) | | (-7.77 - 3.66) | | (-9.66 - 5.79) | (-5.15 - 5.50) |
| Mobile cart share | -6.65 | -0.77 | | -11.04 | | 0.73 | | -13.25* | | -34.37* | -1.67 |
|  | (-22.45 - 9.15) | (-13.76 - 12.22) | | (-30.94 - 8.86) | | (-23.41 - 24.87) | | (-23.37 - -3.13) | | (-60.53 - -8.22) | (-16.51 - 13.17) |
| Stationary cart share | -5.21* | -6.84** | | -3.97 | | -6.98** | | -2.66 | | -1.28 | -5.22* |
|  | (-10.07 - -0.34) | (-11.44 - -2.24) | | (-10.04 - 2.10) | | (-10.24 - -3.71) | | (-9.69 - 4.38) | | (-9.51 - 6.94) | (-10.23 - -0.22) |
| Observations | 12,063 | 4,857 | | 7,206 | | 5,996 | | 6,020 | | 3,575 | 8,488 |
| **B. High blood glucose (%)** | **Total** | | **Male** | | **Female** | | **Low income** | | **High income** | **Sri Lanka** | **Bangladesh** |
| FFR share | 0.06 | | 0.04 | | 0.09 | | 0.06 | | 0.05 | -0.08** | 0.09 |
|  | (-0.03 - 0.15) | | (-0.07 - 0.15) | | (-0.02 - 0.19) | | (-0.06 - 0.18) | | (-0.04 - 0.14) | (-0.13 - -0.02) | (-0.01 - 0.19) |
| Supermarket share | -0.04 | | 0.13 | | -0.33* | | -0.11 | | 0.05 | 0.05 | -0.32 |
|  | (-0.23 - 0.14) | | (-0.13 - 0.40) | | (-0.58 - -0.07) | | (-0.34 - 0.12) | | (-0.13 - 0.23) | (-0.17 - 0.26) | (-0.67 - 0.02) |
| Corner store share | -0.00 | | -0.02 | | 0.01 | | 0.01 | | -0.02 | -0.01 | -0.00 |
|  | (-0.04 - 0.03) | | (-0.06 - 0.02) | | (-0.03 - 0.04) | | (-0.02 - 0.04) | | (-0.07 - 0.02) | (-0.07 - 0.04) | (-0.04 - 0.04) |
| Mobile cart share | -0.11 | | 0.01 | | -0.22** | | -0.05 | | -0.14 | -0.31* | -0.07 |
|  | (-0.26 - 0.05) | | (-0.16 - 0.17) | | (-0.36 - -0.07) | | (-0.29 - 0.19) | | (-0.31 - 0.03) | (-0.54 - -0.07) | (-0.20 - 0.06) |
| Stationary cart share | -0.07 | | -0.08* | | -0.06 | | -0.08* | | -0.05 | 0.01 | -0.07 |
|  | (-0.15 - 0.01) | | (-0.14 - -0.01) | | (-0.16 - 0.04) | | (-0.15 - -0.02) | | (-0.13 - 0.04) | (-0.07 - 0.08) | (-0.13 - 0.00) |
| Observations | 12,050 | | 4,844 | | 7,198 | | 5,992 | | 6,003 | 3,562 | 8,488 |
| **C. Diagnosed DM (%)** | **Total** | | **Male** | | **Female** | | **Low income** | | **High income** | **Sri Lanka** | **Bangladesh** |
| FFR share | 0.09 | | 0.09 | | 0.09 | | 0.05 | | 0.10 | -0.06 | -0.06 |
|  | (-0.02 - 0.19) | | (-0.03 - 0.20) | | (-0.03 - 0.21) | | (-0.06 - 0.16) | | (-0.02 - 0.21) | (-0.18 - 0.06) | (-0.18 - 0.06) |
| Supermarket share | 0.10 | | 0.10 | | 0.08 | | 0.05 | | 0.16 | 0.24* | 0.24* |
|  | (-0.03 - 0.22) | | (-0.13 - 0.34) | | (-0.08 - 0.25) | | (-0.06 - 0.16) | | (-0.04 - 0.36) | (0.06 - 0.42) | (0.06 - 0.42) |
| Corner store share | 0.01 | | 0.00 | | 0.02 | | 0.01 | | 0.02 | 0.02 | 0.02 |
|  | (-0.01 - 0.04) | | (-0.02 - 0.03) | | (-0.01 - 0.05) | | (-0.02 - 0.05) | | (-0.01 - 0.04) | (-0.02 - 0.06) | (-0.02 - 0.06) |
| Mobile cart share | -0.02 | | 0.06 | | -0.10 | | 0.00 | | -0.04 | -0.01 | -0.01 |
|  | (-0.17 - 0.13) | | (-0.09 - 0.20) | | (-0.30 - 0.09) | | (-0.21 - 0.21) | | (-0.14 - 0.07) | (-0.26 - 0.25) | (-0.26 - 0.25) |
| Stationary cart share | -0.02 | | -0.01 | | -0.04 | | -0.04 | | -0.00 | 0.00 | 0.00 |
|  | (-0.09 - 0.04) | | (-0.08 - 0.07) | | (-0.11 - 0.03) | | (-0.10 - 0.02) | | (-0.08 - 0.07) | (-0.09 - 0.10) | (-0.09 - 0.10) |
| Observations | 12,079 | | 4,854 | | 7,217 | | 6,027 | | 6,038 | 3,573 | 3,573 |

*Note: The values show* ***OLS regression coefficients in panel A*** *and* ***average marginal effects (AME****)* ***from logistic regressions in panels B-C****; 95% CIs in brackets. Density/share of outlets= the number of each outlet out of the total number of outlets. For example, supermarket share is defined as the number of supermarkets within a 300m buffer around participant’s home address out of all food outlets within a 300m buffer.* ***No control variables included except for site FE****. All regressions include site fixed effects, where in all regressions we controlled for site specific time invariant characteristics. Level of significance = * p<0∙05, ** p<0∙01.*

**Table A2. UNADJUSTED Associations between food outlet PROXIMITY and fasting blood glucose (OLS coefficients), high blood glucose and diagnosed DM (adjusted odds ratios)**

| **A. Blood glucose level** | **Total** | **Male** | **Female** | **Low income** | **High income** | **Sri Lanka** | **Bangladesh** |
| --- | --- | --- | --- | --- | --- | --- | --- |
| FFR proximity | 1.99* | 3.47* | 0.99 | 0.10 | 3.85** | -0.46 | 2.31 |
|  | (0.12 - 3.86) | (0.41 - 6.52) | (-1.42 - 3.40) | (-3.35 - 3.56) | (1.11 - 6.59) | (-3.92 - 2.99) | (-0.36 - 4.97) |
| Supermarket proximity | -0.94 | 1.86 | -2.42 | 0.02 | -1.54* | 0.28 | -0.36 |
|  | (-3.19 - 1.31) | (-5.54 - 9.26) | (-6.21 - 1.38) | (-6.38 - 6.43) | (-3.01 - -0.06) | (-4.02 - 4.58) | (-1.31 - 0.59) |
| Corner store proximity | 1.40 | 0.03 | 2.40 | 1.97 | 0.58 | -2.00 | 2.69 |
|  | (-1.54 - 4.34) | (-2.94 - 3.00) | (-1.36 - 6.16) | (-1.10 - 5.05) | (-2.61 - 3.77) | (-5.89 - 1.89) | (-1.18 - 6.55) |
| Mobile cart proximity | 1.95 | -0.09 | 3.17 | 3.95 | 1.03 | -0.05 | 2.71* |
|  | (-0.54 - 4.44) | (-4.91 - 4.73) | (-1.30 - 7.64) | (-0.93 - 8.84) | (-2.65 - 4.70) | (-5.82 - 5.72) | (0.43 - 4.99) |
| Stationary cart proximity | 0.73 | 1.16 | 0.26 | 1.63 | -0.54 | 3.40 | -0.06 |
|  | (-2.03 - 3.49) | (-1.79 - 4.11) | (-3.06 - 3.57) | (-2.36 - 5.62) | (-3.95 - 2.87) | (-1.11 - 7.92) | (-3.78 - 3.66) |
| Observations | 12,063 | 4,857 | 7,206 | 5,996 | 6,020 | 3,575 | 8,488 |
| **B. High blood glucose (%)** | **Total** | **Male** | **Female** | **Low income** | **High income** | **Sri Lanka** | **Bangladesh** |
| FFR proximity | 1.15 | 1.30* | 1.06 | 0.98 | 1.31** | 0.81* | 1.31** |
|  | (0.97 - 1.36) | (1.03 - 1.63) | (0.83 - 1.36) | (0.62 - 1.53) | (1.13 - 1.52) | (0.66 - 0.98) | (1.12 - 1.53) |
| Supermarket proximity | 1.01 | 1.22 | 0.90 | 1.00 | 0.99 | 1.06 | 1.12** |
|  | (0.84 - 1.21) | (0.87 - 1.70) | (0.63 - 1.29) | (0.51 - 1.94) | (0.79 - 1.24) | (0.88 - 1.28) | (1.06 - 1.18) |
| Corner store proximity | 1.11 | 1.05 | 1.16 | 1.27 | 0.97 | 0.91 | 1.27 |
|  | (0.92 - 1.34) | (0.80 - 1.37) | (0.94 - 1.44) | (1.00 - 1.61) | (0.75 - 1.27) | (0.74 - 1.12) | (0.99 - 1.63) |
| Mobile cart proximity | 1.02 | 0.91 | 1.09 | 1.06 | 1.02 | 1.00 | 1.01 |
|  | (0.84 - 1.23) | (0.70 - 1.18) | (0.84 - 1.42) | (0.65 - 1.72) | (0.87 - 1.19) | (0.71 - 1.40) | (0.87 - 1.17) |
| Stationary cart proximity | 1.12 | 1.19 | 1.07 | 1.11 | 1.10 | 1.23 | 1.07 |
|  | (0.96 - 1.31) | (0.86 - 1.66) | (0.89 - 1.28) | (0.83 - 1.47) | (0.93 - 1.30) | (0.96 - 1.59) | (0.87 - 1.31) |
| Observations | 12,050 | 4,844 | 7,198 | 5,992 | 6,003 | 3,562 | 8,488 |
| **C. Diagnosed DM (%)** | **Total** | **Male** | **Female** | **Low income** | **High income** | **Sri Lanka** | **Bangladesh** |
| FFR proximity | 1.15 | 1.20 | 1.12 | 1.05 | 1.24** | 0.84 | 1.36** |
|  | (0.96 - 1.37) | (0.94 - 1.55) | (0.90 - 1.39) | (0.77 - 1.42) | (1.08 - 1.42) | (0.64 - 1.12) | (1.15 - 1.60) |
| Supermarket proximity | 1.03 | 1.31** | 0.89 | 0.86 | 1.09 | 1.08 | 1.20** |
|  | (0.74 - 1.44) | (1.07 - 1.60) | (0.55 - 1.44) | (0.45 - 1.65) | (0.83 - 1.44) | (0.67 - 1.74) | (1.11 - 1.29) |
| Corner store proximity | 1.14 | 1.07 | 1.20 | 1.26* | 1.00 | 1.00 | 1.28 |
|  | (0.97 - 1.34) | (0.87 - 1.33) | (0.97 - 1.47) | (1.04 - 1.52) | (0.77 - 1.30) | (0.84 - 1.18) | (1.00 - 1.65) |
| Mobile cart proximity | 1.02 | 0.85 | 1.13 | 1.10 | 0.98 | 1.24 | 0.89 |
|  | (0.78 - 1.32) | (0.63 - 1.14) | (0.79 - 1.60) | (0.84 - 1.43) | (0.72 - 1.33) | (0.98 - 1.58) | (0.69 - 1.14) |
| Stationary cart proximity | 1.15 | 1.31 | 1.04 | 1.13 | 1.14 | 1.02 | 1.20* |
|  | (0.97 - 1.36) | (1.00 - 1.72) | (0.84 - 1.29) | (0.79 - 1.64) | (0.90 - 1.45) | (0.78 - 1.33) | (1.02 - 1.42) |
| Observations | 12,079 | 4,854 | 7,217 | 6,027 | 6,038 | 3,573 | 8,506 |

*Note: The values show* ***OLS regression coefficients in panel A*** *and* ***adjusted odds ratios (AOR) from logistic regressions in panels B-C****; 95% CIs in brackets. Proximity of outlets = 1 if at least one fast food restaurant within 100 m and 0 if otherwise.* ***No control variables included except for site FE****. All regressions include site fixed effects, where in all regressions we controlled for site specific time invariant characteristics. Level of significance = * p<0∙05, ** p<0∙01*
