# Supplementary material for: Food environment and diabetes mellitus in South Asia: A geospatial analysis of health outcome data
Source: PLoS Med. 2022 Apr 26;19(4):e1003970. doi: 10.1371/journal.pmed.1003970 (PMC9041866; doi:10.1371/journal.pmed.1003970)
Supplement: S3 Table — STROBE, Strengthening the Reporting of Observational Studies in Epidemiology. (DOCX) [file pmed.1003970.s006.docx]

**STROBE Statement for cross-sectional studies**

|  | Item No | Recommendation |
| --- | --- | --- |
| **Title and abstract** | 1 | (*a*) Indicate the study’s design with a commonly used term in the title or the abstract  Yes, in the title and Abstract. |
|  |  | (*b*) Provide in the abstract an informative and balanced summary of what was done and what was found  Yes, Methods and Findings. |
| Introduction | | |
| Background/rationale | 2 | Explain the scientific background and rationale for the investigation being reported  Yes, Introduction, paragraphs 1-4 |
| Objectives | 3 | State specific objectives, including any prespecified hypotheses  Yes, Introduction, paragraph 4 |
| Methods | | |
| Study design | 4 | Present key elements of study design early in the paper  Yes, Methods, paragraph 1 |
| Setting | 5 | Describe the setting, locations, and relevant dates, including periods of recruitment, exposure, follow-up, and data collection  Yes, Methods, paragraphs 1-3 |
| Participants | 6 | (*a*) Give the eligibility criteria, and the sources and methods of selection of participants  Yes, Methods, paragraph 2 |
| Variables | 7 | Clearly define all outcomes, exposures, predictors, potential confounders, and effect modifiers. Give diagnostic criteria, if applicable  Yes, Methods, paragraphs 3-10 |
| Data sources/ measurement | 8* | For each variable of interest, give sources of data and details of methods of assessment (measurement). Describe comparability of assessment methods if there is more than one group  Yes, Methods paragraph 3 for outcome variables and paragraph 5 for independent variables |
| Bias | 9 | Describe any efforts to address potential sources of bias  Yes, Methods, paragraph 8 |
| Study size | 10 | Explain how the study size was arrived at  Yes, Methods, paragraphs 1-2 |
| Quantitative variables | 11 | Explain how quantitative variables were handled in the analyses. If applicable, describe which groupings were chosen and why  Yes, Methods paragraph 3 for outcome variables and paragraph 5 for independent variables |
| Statistical methods | 12 | (*a*) Describe all statistical methods, including those used to control for confounding  Yes, Methods, paragraphs 7-9 |
|  |  | (*b*) Describe any methods used to examine subgroups and interactions  Yes, Methods, paragraph 8 |
|  |  | (*c*) Explain how missing data were addressed  We only used non-missing data |
|  |  | (*d*) If applicable, describe analytical methods taking account of sampling strategy  Not applicable |
|  |  | (*e*) Describe any sensitivity analyses  Not applicable |
| Results | | |
| Participants | 13* | (a) Report numbers of individuals at each stage of study—eg numbers potentially eligible, examined for eligibility, confirmed eligible, included in the study, completing follow-up, and analysed  Yes, Methods, paragraphs 1-2. Also, Appendix 3 for CONSORT diagram. |
|  |  | (b) Give reasons for non-participation at each stage  Yes, Appendix 3 CONSORT diagram. |
|  |  | (c) Consider use of a flow diagram  Yes, Appendix 3 CONSORT diagram. |
| Descriptive data | 14* | (a) Give characteristics of study participants (eg demographic, clinical, social) and information on exposures and potential confounders  Yes, Table 1 |
|  |  | (b) Indicate number of participants with missing data for each variable of interest  Yes, see Appendix 3 CONSORT diagram. |
| Outcome data | 15* | Report numbers of outcome events or summary measures  Yes, Table 1 |
| Main results | 16 | (*a*) Give unadjusted estimates and, if applicable, confounder-adjusted estimates and their precision (eg, 95% confidence interval). Make clear which confounders were adjusted for and why they were included (YES)  Yes, unadjusted estimates were provided in the Appendix |
|  |  | (*b*) Report category boundaries when continuous variables were categorized  Yes, Methods, paragraph 7 |
|  |  | (*c*) If relevant, consider translating estimates of relative risk into absolute risk for a meaningful time period  Not applicable |
| Other analyses | 17 | Report other analyses done—eg analyses of subgroups and interactions, and sensitivity analyses  Yes, Tables 2-3 and Figure 1-3 |
| Discussion | | |
| Key results | 18 | Summarise key results with reference to study objectives  Yes, Discussion, paragraphs 1-3 |
| Limitations | 19 | Discuss limitations of the study, taking into account sources of potential bias or imprecision. Discuss both direction and magnitude of any potential bias  Yes, Discussion, paragraph 12 |
| Interpretation | 20 | Give a cautious overall interpretation of results considering objectives, limitations, multiplicity of analyses, results from similar studies, and other relevant evidence  Yes, Discussion, paragraph 12 |
| Generalisability | 21 | Discuss the generalisability (external validity) of the study results  Yes, Discussion, paragraph 11 |
| Other information | | |
| Funding | 22 | Give the source of funding and the role of the funders for the present study and, if applicable, for the original study on which the present article is based  Yes in Acknowledgement |

*Give information separately for exposed and unexposed groups.
